# Beneficial Role of Rosuvastatin in Blood–Brain Barrier Damage Following Experimental Ischemic Stroke

**DOI:** 10.3389/fphar.2018.00926

**Published:** 2018-08-21

**Authors:** Dan Lu, Hong-Cheng Mai, Yu-Bin Liang, Bing-Dong Xu, An-Ding Xu, Yu-Sheng Zhang

**Affiliations:** ^1^Department of Neurology and Stroke Center, The First Affiliated Hospital, Jinan University, Guangzhou, China; ^2^Clinical Neuroscience Institute of Jinan University, Jinan University, Guangzhou, China

**Keywords:** tissue type plasminogen activator therapy, blood–brain barrier, rosuvastatin, tight junction protein, matrix metalloproteinase, focal cerebral ischemia

## Abstract

Hemorrhage transformation is the most challenging preventable complication in thrombolytic therapy and is related to recombinant tissue plasminogen activator (rt-PA)-induced blood–brain barrier (BBB) damage. Intraperitoneal injections of normal or high doses of rosuvastatin were administered to Balb/c mice 20 min prior to middle cerebral artery occlusion (MCAO) surgery for 3 h followed by reperfusion with rt-PA thrombolytic therapy and cerebral blood flow monitoring to investigate whether a high or normal dose of rosuvastatin reduces BBB damage after brain ischemia and rt-PA reperfusion. The integrity of the BBB was ameliorated by normal and high doses of rosuvastatin as determined from Evans blue staining, ultrastructure assessments and immunochemistry at 24 h after reperfusion. The levels of TJ proteins were preserved, potentially by targeting platelet-derived growth factor receptor α (PDGFR-α) and low-density lipoprotein receptor-related protein 1 (LRP1) to inhibit the expression of matrix metalloproteinase proteins (MMPs) by reducing the levels of phosphorylated c-jun-N-terminal kinase (pJNK), phosphorylated mitogen-activated protein kinase (MAPK) p38 (pP38) and increasing the levels of phosphorylated extracellular regulated protein kinases (pERK), and tissue inhibitor of metalloproteinases (TIMPs), as inferred from Western blotting and molecular docking analyses. In summary, rosuvastatin reduced rt-PA therapy-associated BBB permeability by PDGFR-α- and LRP1-associated MAPK pathways to reduce the mortality of mice, and a normal dose of rosuvastatin exerted greater preventative effects on reducing BBB damage than did a high dose in the time window of thrombolytic therapy.

## Introduction

Intravenous thrombolytic therapy with rt-PA within 3 h after symptom onset in patients with AIS is beneficial and has been widely recommended in clinical practice guidelines (National Institute of Neurological Disorders and Stroke rt-PA Stroke Study Group, 1995; Saver et al., 2015). Unfortunately, HT is the most worrisome complication after thrombolytic therapy. Specifically, 4.4 ∼ 6.8% (Emberson et al., 2014; Goyal et al., 2016) of sICHs occur after intravenous thrombolytic therapy with rt-PA for AIS. Craniectomy control was used to prevent brain hernia in patients with severe hematomas (Fan et al., 2014). Agents that reverse coagulopathy (cryoprecipitate, platelet transfusion, prothrombin complex concentrate, fresh-frozen plasma, vitamin K, antifibrinolytic agents, and activated factor VII) have been used to decrease further HT-associated bleeding based on expert opinion and a small case series, but the efficacy and safety of these treatments are not well established (Yaghi et al., 2017).

Structurally, the opening of the BBB is associated with rt-PA, which functions as a signaling molecule and mediates MMP activation in early HT (Khatri et al., 2012; Jickling et al., 2014; Chang et al., 2017). Neuroprotective agents, such as free radical scavengers, MMP inhibitors, LRP antagonists, platelet inhibitors, albumin, annexin 2, activated protein C, estrogen, neuroserpin, and tacrolimus, have been shown to reduce the vascular damage of HT when combined with tissue plasminogen activator in animal models, potentially by decreasing MMP expression and activity (Suzuki et al., 2011; Jickling et al., 2014; Turner and Sharp, 2016; Knecht et al., 2017). However, clinical therapies targeting these molecular mediators of rt-PA-induced vascular damage are still under development.

Statins, also known as HMG-CoA reductase inhibitors, are a class of common clinical lipid-lowering medications. Overwhelming evidence shows that statins are safe and effective for the primary and secondary prevention of ischemic stroke caused by atherosclerosis (Goldstein et al., 2008). Recently, THRombolysis and STatins (THRaST) studies (Erdur et al., 2018) have consistently shown that high-dose statin use in the acute phase of stroke after intravenous thrombolysis has positive effects on short- and long-term outcomes (Cappellari et al., 2013). Moreover, a lower risk of sICH was detected in a statin group than in a non-statin group, although the difference between the two groups was not significant (1.2% vs. 3.8%, *P* = 0.176) (Cappellari et al., 2013). The pleiotropic effects might be associated with the statin dosage. There has been considerable debate regarding the most effective dose of rosuvastatin, which is the most commonly used statin in clinical treatment, for BBB conservation during the acute phase of a stroke after intravenous thrombolysis.

The current study investigated whether a high or normal dose of rosuvastatin reduced BBB damage following rt-PA reperfusion stroke-related hemorrhage in the MCAO mouse model, thereby reducing mortality, and it also aimed to identify the potential mechanisms underlying the protective effects on the BBB following MCAO in mice followed by rt-PA reperfusion.

## Materials and Methods

### Experimental Design

A total of 228 specific pathogen-free male Balb/c mice (12 weeks old, 22–25 g) were used for this study, which was expanded on a previous study (Lu et al., 2018). 169 of the animal samples obtained from the previous study (Lu et al., 2018) were used in experiments of mortality, TTC staining, electron microscopy, assessments of BBB integrity, immunofluorescence staining and Western blotting in this study. This study was carried out in accordance with the recommendations of the NIH Guide (NIH Publications No. 8023, revised 1978) for the Care and Use of Laboratory Animals. The protocol was approved by the Competent Ethics Committees of Jinan University (Certificate Number of Laboratory Animal Ethics: 20160118232728) and aimed to reduce the total number of animals and their potential pain and suffering.

**FIGURE 1:**
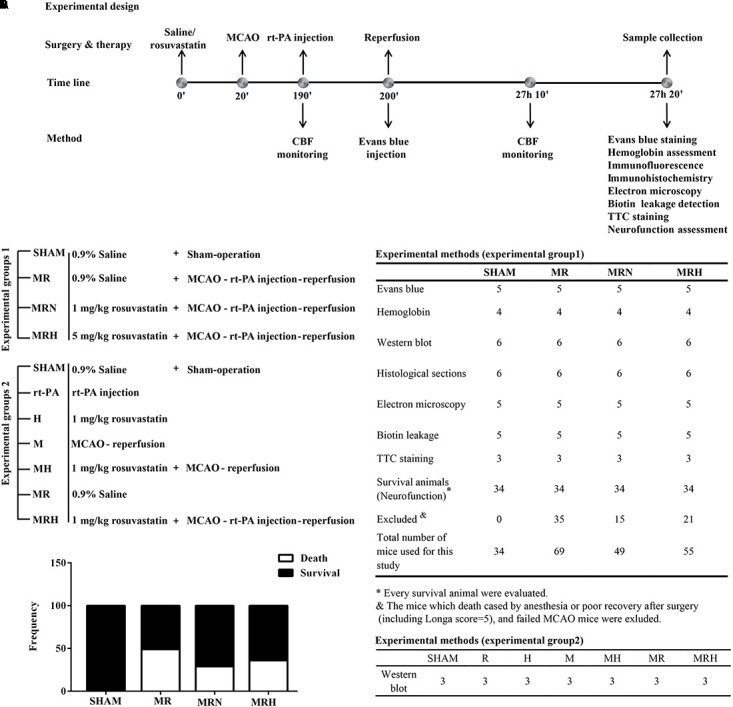
Schematic overview of the protocols. **(A)** Illustration of the protocols, including the time line of the experiments and tests. **(B)** The treatment groups of experiments 1 and 2. **(C)** The tests and numbers of mice in experiments 1 and 2. **(D)** The frequencies of death and survival in mice, which were analyzed by chi-square test.

Experiment 1 (**Figure 1B**) was designed to evaluate whether pretreatment with a normal or high dose of rosuvastatin reduced rt-PA reperfusion/stroke-related mortality within 24 h (primary endpoint). This experiment involved four groups (**Figures 1B,C**). The secondary endpoints were focal stroke deficits (focal neurological deficits) and hemorrhage effects (**Figure 1C**). Two hundred and seven mice were randomly divided into four groups of *n* = 34 mice each: (1) a sham-operated group (SHAM), (2) mice that underwent MCAO for 3 h followed by rt-PA (Actilyse, Boehringer Ingelheim Pharma GmbH & Co., United Kingdom) reperfusion for 24 h (MR), (3) mice pretreated with a normal dose of rosuvastatin (1 mg/kg, dissolved in normal saline; Sigma-Aldrich-Fluka, MO, United States), equivalent to the clinical application of a 20-mg normal-dose oral treatment to a 70-kg adult for the secondary prevention of cerebrovascular disease) prior to MCAO for 3 h followed by rt-PA reperfusion for 24 h (MRN), and (4) mice pretreated with a high dose of rosuvastatin (5 mg/kg, equivalent to the clinical application of an 80-mg high-dose oral treatment to a 70-kg adult for the secondary prevention of cerebrovascular diseases) prior to MCAO for 3 h followed by rt-PA reperfusion for 24 h (MRH) (Jones et al., 2003; Goldstein et al., 2009; Peng et al., 2009). Due to death by anesthesia or poor recovery after surgery (including mice with a Longa score = 5), 28 mice were excluded. In addition, 43 mice with a failed MCAO were excluded from the experiment.

Experiment 2 involved seven groups (**Figures 1B,C**) and was designed to investigate the mechanism underlying the protective effects of rosuvastatin. Twenty-one mice were divided into seven groups (*n* = 3 mice per group): (1) a sham-operated group (SHAM), (2) mice receiving a normal dose of rosuvastatin (1 mg/kg) for 24 h (N), (3) mice receiving rt-PA for 24 h (R), (4) mice that underwent MCAO for 3 h followed by reperfusion for 24 h (M), (5) mice receiving a normal dose of rosuvastatin followed by MCAO for 3 h and reperfusion for 24 h (MN), (6) mice that underwent MCAO for 3 h followed by rt-PA reperfusion for 24 h (MR), and (7) mice pretreated with a normal dose of rosuvastatin prior to MCAO for 3 h followed by rt-PA reperfusion for 24 h (MRN).

### Experimental Models and Laser Speckle Perfusion Imaging

The MCAO model was established using an intraluminal filament as previously described (Cai et al., 2017). Mice were anesthetized with isoflurane (4% for induction anesthesia, 1.5% for maintenance anesthesia; RWD Life Science, Shenzhen, China) in air. The temperature was maintained at 37.0 ± 0.5°C using a heating pad, and focal ischemia was induced using an intraluminal filament technique. A midline incision was made in the neck, and the left common carotid artery (CCA), external carotid artery and internal carotid artery were isolated. Briefly, the stump of the external carotid artery was cut; then, a filament made of 8–0 nylon string coated with silicon (RWD Life Science) was carefully inserted into the internal carotid artery and advanced along to the internal carotid artery 11 mm from the carotid artery bifurcation or until resistance was encountered. Three hours after the induction of MCAO, thrombolysis was conducted by administering rt-PA (10 mg/kg) (Niego et al., 2017) in saline via the tail vein for 10 min, and the filament was carefully withdrawn to induce vascular recanalization/reperfusion for 24 h. Then, the mice were kept in a cage and allowed free access to food and water. The SHAM group also received the surgery but without filaments. A successful model was achieved when there was an approximately 80% reduction in cerebral blood flow (CBF), which was determined using a blood perfusion imager (PeriCam PSI System, Perimed AB, Stockholm, Sweden) and laser speckle contrast analysis (LASCA) technology as described previously (Pfeilschifter et al., 2011). Relative CBF (rCBF) values were obtained by normalizing to the SHAM group (**Figure 1A**).

### TTC (2, 3, 5-Triphenyltetrazolium Chloride) Staining

Three mice in each group were deeply anesthetized and transcardially perfused with cold saline 24 h after rt-PA reperfusion to determine the sizes of the infarct and surrounding hemorrhage site before tissue collection. The brains were quickly removed and sectioned coronally into 2-mm slices for TTC staining. Before being imaged with a Canon camera, slices were stained with 2% TTC and incubated in a dark chamber at 37°C for 20 min. Hemispheric volumes were captured by camera (Simao et al., 2017).

### Evans Blue Staining

Alterations in brain vascular permeability were determined by tail vein injections of Evans blue dye (Sigma-Aldrich). Evans blue binds to serum proteins such as albumin and can be used to quantify alterations in vascular permeability since albumin does not cross the endothelial barrier under basal physiological conditions (Khan et al., 2011). Briefly, five mice were injected with 2% Evans blue solution in normal saline (4 ml/kg of body weight) 24 h before reperfusion. The Evans blue solution was allowed to circulate for 24 h. Six mice were euthanized and perfused with 50 ml of ice-cold phosphate buffered saline (PBS). The brains were then removed and divided into ipsilateral and contralateral hemispheres. Evans blue was extracted at 55°C overnight with formamide. Dye concentrations in the supernatant were quantified spectrophotometrically at 620 nm and normalized to hemisphere weight (Chang et al., 2017).

### Spectrophotometric Assay for Hemoglobin

The hemoglobin content was quantified with a spectrophotometric assay (Jia et al., 2010). Three mice selected randomly from each group were perfused transcardially with cold saline. The brains were quickly removed to be processed for the detection of hemoglobin. The left hemisphere brain tissue was homogenized by adding 10 ml/g saline and centrifuging at 13,000 rpm for 30 min. Then, the supernatant containing hemoglobin was collected. A total of 80 μl of Drabkin reagent (Sigma) was added to 200 μl of supernatant. After the samples (the detection was repeated three times for each mouse) were left for 15 min at room temperature, the optical density in each group was measured at 540 nm with a spectrophotometer (Varioskan LUX; Thermo Fisher Scientific, MA, United States). Based on the standard absorbance curve, the measured optical density absorption was converted to hemoglobin volume. The total hemoglobin content of each group is expressed as micrograms per sample.

### Leakage of EZ-Link Sulfo-NHS-Biotin for BBB Integrity Determination

Five mice in each group were deeply anesthetized with isoflurane. A total of 50 ml of 0.01 mol/l PBS containing EZ-link Sulfo-NHS-Biotin (0.5 mg/ml, cat. # 21217, Thermo Scientific) was perfused into the beating heart via the left ventricle over the course of 5 min, following by the perfusion of 50 ml ice-cold 1% paraformaldehyde (PFA) diluted in 0.01 mol/l PBS. Brains were dissected and fixed in 4% PFA for 1 h at room temperature. Brains were cut into 2-mm-thick slices and transferred to 30% sucrose in distilled water overnight at 4°C before embedding in OCT. Frozen sections 10-μm thick were allowed to dry on adhesive microscope slides (CITOGLAS) at room temperature for 1 h before being rehydrated in 0.01 mol/l PBS. Tissue samples were then blocked in 0.01 mol/l PBS + 10% normal goat serum + 0.3% Triton X-100 for 30 min at room temperature. Slides were incubated at 4°C with the following primary antibodies in 0.01 mol/l PBS + 5% normal goat serum + 0.3% Triton X-100: sheep anti-mouse vWF (1:200, Abcam, Billerica, MA, United States) and FITC-streptavidin. Slides were washed in 0.01 mol/l PBS for 5 min twice remove excess antibody. After secondary fluorescently labeled antibodies (Cy3 rabbit anti-sheep IgG, diluted 1:250 in 0.01 mol/l PBS + 5% normal goat serum + 0.3% Triton X-100) were incubated with the samples at room temperature for 1 h, DAPI (Vector Labs, Burlingame, CA, United States) was used to label nuclei. Before the glass slides were coverslipped using mounting medium, excess antibodies were removed by washing the slides twice with 0.01 mol/l PBS for 3 min each.

### Histological Sections

Six mice selected randomly from each group were perfused transcardially with ice-cold 0.9% saline followed by 4% PFA in PBS (0.1 mol/l, pH 7.4). Brains were dissected and fixed in 4% PFA for 1 h at room temperature. Brains were cut into 2-mm-thick slices and transferred to 30% sucrose in distilled water overnight at 4°C before being embedding in optimal cutting temperature compound. Coronal sections (10-μm thick) were cut with a cryostat (CM1900, Leica, Heidelberger, Germany) and stored at -80°C until used for Perls’ iron staining, immunohistochemistry and immunofluorescence.

### Perls’ Iron Staining

Perls’ iron staining was used as previously described to observe local hemorrhagic lesions. Briefly, sections were incubated in Perls’ iron staining solution [1% K_4_Fe (CN)_6_ and 1% HCl] for 30 min and then counterstained with Neutral Red counterstain for 5–10 s (Nakamura et al., 2003).

### Immunofluorescence

Immunofluorescence staining was performed on sections incubated with primary antibodies against matrix metalloproteinase-2 (MMP-2, 1:100), matrix metalloproteinase-9 (MMP-9, 1:50), at 4°C overnight for 16 h. Then, sections were incubated with appropriate fluorescence-labeled secondary antibodies at room temperature for 1 h [Alexa Fluor 594 goat polyclonal anti-mouse IgG H&L and FITC 488-conjugated goat polyclonal anti-rabbit IgG H&L (1:500, YEASEN, China)]. MMP-2 (green) and MMP-9 (red) levels were assessed in the whole slide, and the peri-infarct areas underwent image analysis using the Image-Pro Plus 6.0 software (Media Cybernetics, Inc., United States). Red or green granules in the cytoplasm were considered to be positive immunostaining. The number of the positive cells was measured and normalized based on DAPI staining.

### Electron Microscopy

Five mice selected from each group were perfused transcardially with a cold 0.1 mol/l PBS solution followed by 2.5% glutaraldehyde. The brains were then removed and dissected for electron microscopy. The brain sections were dehydrated, embedded in epoxy resin, cut and visualized with a HITACHI Transmission Electron Microscope (HITACHI, Tokyo, Japan) at 80 kV. Moreover, sections were selected according to the described groups, and five areas in the ipsilateral peri-infarct cortex in each section were chosen.

### Western Blotting

Brains from six mice in experiment 1 and three mice in experiment 2 were quickly removed and stored at -80°C until Western blot analysis. Proteins (8 μg) from peri-infarcted brain tissues were immunoblotted to detect vascular endothelial cadherin (VE-cadherin, located at the intercellular junctions of endothelial cells, where it is thought to play a role in the cohesion and organization of intercellular junctions; 1:1000, Cell Signaling Technology, Danvers, MA, United States); claudin-5 (integral membrane proteins and components of TJ strands located in both epithelial and endothelial cells in all TJ-bearing tissues; 1:1000, Wanleibio, Shenyang, China); MMP-1 (1:1000, Cell Signaling Technology); MMP-2 (one of the ubiquitous metalloproteinases involved in diverse functions, such as vascular remodeling, angiogenesis, and tissue repair; 1:1000, Abcam); MMP-3 (degrades fibronectin; laminin; type I, III, IV, and V gelatins; collagens III, IV, X, and IX; and cartilage proteoglycans; 1:1000, Abcam); MMP-9 (an important factor in normal tissue remodeling during wound healing, angiogenesis, and apoptosis; 1:1000, Wanleibio); occludin (a tetraspan integral membrane protein in epithelial and endothelial TJ structures that comprises two extracellular loops; 1:1000, Wanleibio); tissue inhibitor of metalloproteinases 1 (TIMP-1; 1:1000, Wanleibio); TIMP-2 (1:1000, Wanleibio); GFAP (1:1000, Abcam); low-density lipoprotein receptor-related protein 1 (LRP-1; 1:20000, Abcam); PDGFR-α (1:1000, Elabscience, Wuhan, China); low-density lipoprotein receptor (LDLr; 1:1000, Abcam); phospho-NF-κB p65 (pNFκB; 1:1000, Cell Signaling Technology), pJNK (1:1000, Cell Signaling Technology), phosphorylated mitogen-activated protein kinase (MAPK) p38 (pP38; 1:1000, Cell Signaling Technology), pERK (1:1000, Cell Signaling Technology) and β-actin (1:3000, Cell Signaling Technology). After incubation with the diluted primary antibodies and appropriate horseradish peroxidase-conjugated secondary antibodies (Amersham Biosciences, Buckinghamshire, United Kingdom), immunodetection was performed using an enhanced chemiluminescent substrate (Millipore, Massachusetts, United States). β-Actin (1:20000; Sigma) was used as a loading control for protein quantification. The density of the protein bands was exposed and analyzed with a Tanon 2500 Gel Imaging System (Tanon, Shanghai, China).

### Protein–Protein Interaction Network Construction

The Search Tool for the Retrieval of Interacting Genes (STRING) database aims to construct functional protein association networks by consolidating known and predicted protein–protein association data for a large number of organisms (Szklarczyk et al., 2017). The STRING resource is available at http://string-db.org/. The corresponding protein-protein interaction networks for LRP1, PDGFR-α, and the MMPs and TIMPs were constructed by selecting the interactions pertaining to Homo sapiens that showed a minimum interaction confidence score >0.9.

### Molecular Docking

The rosuvastatin ligands were identified using X-ray techniques and 2D-NMR based on the PubChem compound^1^. Then, rosuvastatin was evaluated for its binding energies with PDGFR-α, LRP-1, and LDLr. PDB files of proteins were downloaded from the RCSB Protein Data Bank^2^. Molecular docking was carried out with Auto-Dock Vina (The Scripps Research Institute, La Jolla, CA, United States) following a previously reported protocol (Hong et al., 2017). Discovery Studio 4.5 (BIOVIA, Beijing, China) was used for the visualization of the binding modes obtained from the docking. The inhibition constant for a drug against a target protein can be operationalized by the formula:


$$\begin{array}{c} Ki\;=\;\exp[(\Delta G\;\times \;1000)/(\mathrm{Rcal}\;\times \;T)]. \end{array}$$

In the formula, affinity is ΔG, with a unit of kcal⋅mol^-1^; T = 300 K; and Rcal = 1.98719 cal⋅mol^-1^⋅K^-1^.

### Statistical Analysis

SPSS 19.0 software (IBM, United States) was used for statistical analyses. Data are presented as the means ± SEM (standard errors of the means). One-way ANOVA was used for comparisons among multiple groups, followed by a multiple comparison LSD test with the baseline control. A chi-square or Fisher exact test was used to compare rates. Statistical significance was set at the level of *P* < 0.05. The Kruskal–Wallis test and Wilcoxon non-parametric test were used to compare the Longa scores among the groups at a significance level of 0.05.

## Results

### Rosuvastatin Decreased BBB Permeability at 24 h Following rt-PA Reperfusion After Brain Ischemia

**FIGURE 2:**
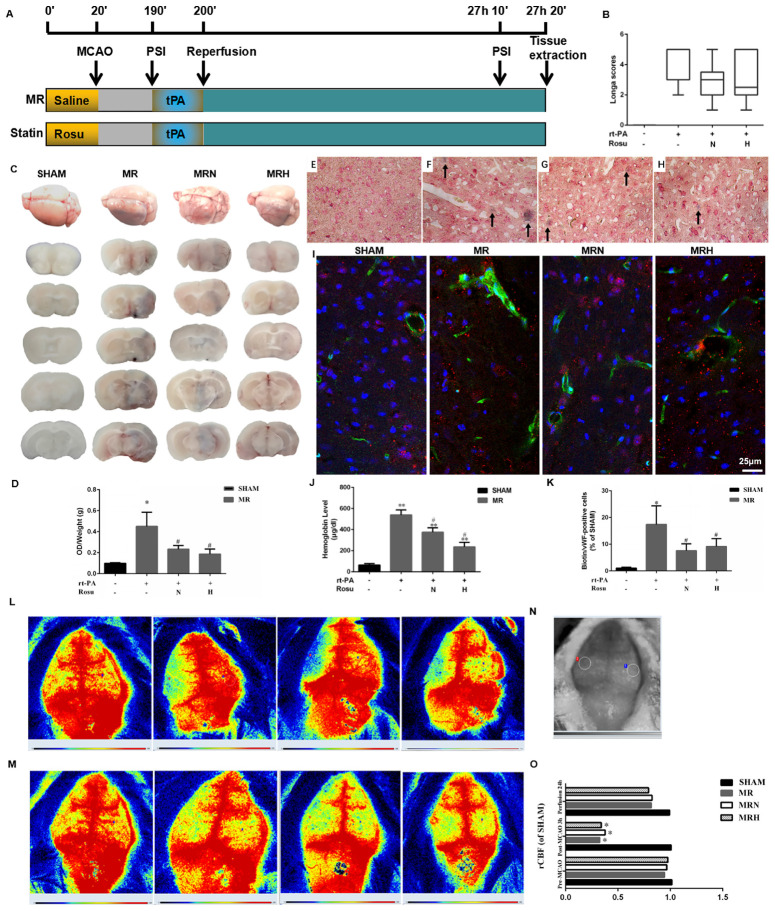
Rosuvastatin decreased BBB permeability at 24 h following rt-PA reperfusion after brain ischemia but did not alter cerebral blood reflow. **(A)** Experimental timeline showing MCAO, rosuvastatin/saline administration, rt-PA reperfusion, laser speckle imaging, and tissue collection. **(B)** Neurological deficits were evaluated in a blinded manner by determining the Longa score before MCAO and at 24 h after reperfusion in the SHAM, MR, MRN, and MRH groups. **(C)** Representative whole-brain and coronal brain sections from the SHAM, MR, MRN, and MRH groups after MCAO followed by 24 h reperfusion. The dark blue color indicates Evans blue-stained leakage areas. **(D)** Evans blue leakage in the brain was quantified spectrophotometrically and normalized to brain tissue weight. **(E–H)** Representative Perls’ iron staining images showing microvascular hemorrhage in the SHAM, MR, MRN, and MRH groups, respectively. The dark blue areas indicated by black arrows are associated with hemorrhage. Scale bar, 50 μm. **(I)** Representative images showing the Sulfo-NHS-Biotin tracer extravasation assay. Biotin leakage is shown in green, and vWF-positive vessels are shown in red. Scale bar, 25 μm. **(J)** Quantitative analysis of intracerebral hemorrhage using a hemoglobin assay. **(K)** Quantitative analysis of biotin/vWF-positive cells normalized to the SHAM group. **(L)** Representative cerebral blood flow images before reperfusion in the SHAM, MR, MRN, and MRH groups captured by 2-D laser speckle imaging. **(M)** Representative cerebral blood flow images after 24 h reperfusion in the SHAM, MR, MRN, and MRH groups captured by 2-D laser speckle imaging. **(N)** Circles indicate the monitored areas for cerebral blood flow analysis. **(O)** Quantitative analysis of relative cerebral blood flow. Data are presented as mean ± SEM. *P < 0.05 and ^**^P < 0.01 compared with SHAM; ^#^*P* < 0.05 compared with MR; ^&^*P* < 0.05 compared with MRN. MR, mice that underwent MCAO for 3 h followed by rt-PA reperfusion for 24 h; MRN, mice pretreated with a normal dose of rosuvastatin (1 mg/kg) prior to MCAO for 3 h followed by rt-PA reperfusion for 24 h; MRH, mice pretreated with a high dose of rosuvastatin (5 mg/kg) prior to MCAO for 3 h followed by rt-PA reperfusion for 24 h.

The mortality and survival rates were calculated as frequencies and analyzed using the chi-square test, which showed significant differences among the MR, MRN, and MRH groups (*P* = 0.013, chi-square test). However, there was no significant difference in neurological score between the normal-dose and high-dose rosuvastatin therapy groups (*P* = 0.365, Fisher’s exact test) (**Figure 1D**). We monitored CBF at three time points, i.e., before MCAO, after ischemia (**Figure 2A**) and at 24 h after rt-PA reperfusion (**Figure 2B**), using a two-dimensional laser speckle imaging technique. Laser speckling was used to generate whole-brain images of blood flow to demonstrate the changes in blood flow over the entire ischemic territory (**Figure 2C**). Mice with statin therapy subjected to MCAO demonstrated similar decreases to those of the MCAO group; after rt-PA reperfusion and filament withdrawal recanalization at 24 h, mice subjected to MCAO (with rosuvastatin) demonstrated similar extents of recovery from ischemia as exhibited by mice in the MR group (**Figure 2D**).

One of the secondary endpoints, neurological deficits (**Figure 2E**), was evaluated to assess the outcomes of brain ischemia in mice. The mice in the SHAM group did not display any signs of neurological deficits or mortality. Twenty-four hours after reperfusion following MCAO, mice in the MR group showed greater neurological deficits (Longa scores of 3.63 ± 1.29 and 3.95 ± 1.16 for the normal-dose and high-dose groups, respectively) than did mice in the SHAM group (Longa score 0, *P* < 0.01, Kruskal–Wallis test). No significant difference in neurological scores was observed between the MR group and both the normal-dose (3.08 ± 1.28) or high-dose (3.04 ± 1.48) rosuvastatin therapy groups (*P* > 0.05, Wilcoxon non-parametric test).

To analyze the brain hemorrhaging caused by BBB damage, Evans blue staining, Perls’ iron staining and spectrophotometric hemoglobin assays were performed on SHAM (sham-operated mice), MR (mice with rt-PA reperfusion 24 h after MCAO), MRN (mice with normal-dose rosuvastatin therapy and rt-PA reperfusion 24 h after MCAO), and MRH (mice with high-dose rosuvastatin therapy and rt-PA reperfusion 24 h after MCAO) groups. To determine if rosuvastatin affected the integrity of the BBB, we injected Evans blue solution and examined its extravasation into the brain. Brain samples were coronally sliced into ∼2-mm sections, and the amount of blue dye leakage from the BBB was assessed as an indicator of the severity of the BBB disruption in the groups with rt-PA reperfusion 24 h after MCAO (**Figure 2F**). Mice treated with both normal and high doses of rosuvastatin were significantly protected against BBB permeability following rt-PA reperfusion 24 h after MCAO. Minimal Evans blue extravasation was observed in the SHAM group (**Figure 2G**). The decreased permeability observed in the statin-treated mice that received rt-PA reperfusion 24 h after MCAO was further confirmed by Perls’ iron staining (**Figure 2H**) and spectrophotometric hemoglobin assays (**Figure 2K**). Morphological analysis based on Perls’ iron staining revealed distinct blue-stained clusters in the intralesional and perilesional areas of mice that had undergone rt-PA reperfusion 24 h after MCAO, but not in the SHAM group; however, lower levels of blue-stained clusters were observed in the normal-dose and high-dose rosuvastatin therapy groups than in the mice that only received rt-PA reperfusion after MCAO (**Figure 2H**). In addition, increased leakage of the exogenous tracer Sulfo-NHS-Biotin, as an indicator of BBB integrity, from vWF-positive cells was observed in MCAO mice after rt-PA reperfusion. In contrast, lower leakage of Sulfo-NHS-Biotin from vWF-positive cells was observed in the statin therapy groups, indicating a reduction in BBB disruption (**Figures 2I,J**). In agreement with the findings based on Perls’ iron staining and biotin leakage, the mean hemoglobin content was significantly higher in the MR group at 24 h after rt-PA reperfusion following MCAO than in the SHAM group. Importantly, significantly lower mean hemoglobin contents were observed in the normal- and high-dose statin therapy groups than in the non-statin therapy group that received rt-PA reperfusion 24 h after MCAO (**Figure 2K**; *P* < 0.05).

### Rosuvastatin Upregulated the Levels of Tight Junctions (TJs) and Adherence Junctions (AJs) in the Peri-Infarct Region Following Reperfusion

**FIGURE 3:**
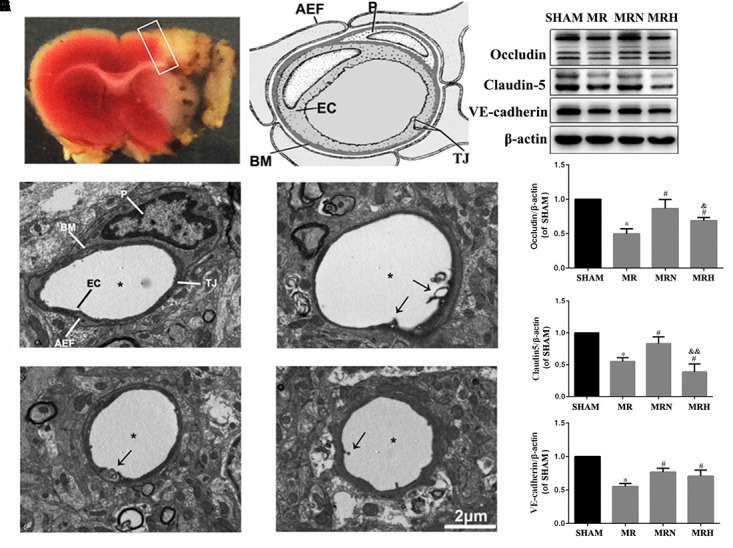
Rosuvastatin upregulated the levels of TJs and AJs in the peri-infarct region following reperfusion. **(A)** Representative coronal sections stained by TTC following MCAO and after 24 h reperfusion. The white color indicates the infarct region, which was observed as a pale color with the naked eye, and the white box indicates the peri-infarct region being observed. **(B)** General view of neurovascular unit is presented; P indicates pericyte, EC indicates endothelial cell, AEF indicates astrocytic end-feet, TJ indicates tight junction. The images were visualized using TEM at a magnification of 6000× (scale bar = 2 μm). **(C)** Transmission electron micrographs show the normal ultrastructure of the BBB in the cerebral peri-infarct area, which is labeled similarly to the general view of a neurovascular unit seen in **(B)**. **(D)** Transmission electron micrographs show the abnormal ultrastructure of the BBB in the cerebral peri-infarct area of MR mice. Stars indicate the microvessel lumen, and arrows indicate broken TJs. **(E,F)** show the abnormal ultrastructure of the BBB in the cerebral peri-infarct areas of MRN and MRH mice under TEM, respectively. Stars indicate the microvessel lumen, and the arrows indicated broken TJs. **(G)** The bands for the occludin, claudin-5, VE-cadherin, and β-actin proteins were detected by Western blotting. **(H–J)** The relative densities were assessed by calculating the ratios of the occludin, claudin-5 and VE-cadherin proteins to β-actin and compared with those of the SHAM group (^∗^*P* < 0.05), among the MRN, MRH, and MR groups (^#^*P* < 0.05), and between the MRN and MRH groups (^&^*P* < 0.05, ^&&^*P* < 0.01). Means ± SEM, *n* = 6.

As part of the neurovascular unit, the endothelial layer and interendothelial belts of TJs are thought to prevent increases in BBB permeability. The cortical capillaries in the SHAM group showed a normal ultrastructure, with a single layer of endothelial cells (ECs) along with smooth microvessels and abundant and intact TJ complexes, surrounded by a complete basement membrane (BM) and pericyte and astrocyte end-feet (**Figures 3B,C** and **Supplementary Figure S1A**). Compared with that in the SHAM group, the capillary ultrastructure in the ipsilateral cortex showed various abnormalities in the peri-infarct regions of the MR group (**Figure 3A**), including edematous ECs and pericytes, disrupted BMs, unclear and disjointed TJs, and swollen astrocyte end-feet (**Figure 3D** and **Supplementary Figure S1B**). The rosuvastatin therapy groups showed an attenuation of these various morphological abnormalities caused by rt-PA reperfusion 24 h after 3 h of MCAO (**Figures 3E,F** and **Supplementary Figures S1C,D**).

To further investigate the protein level alterations of TJs and AJs, we evaluated the expression of claudin-5, VE-cadherin, and occludin via Western blotting (**Figure 3G**). The proportion of occludin in the MR group was significantly lower than that of the SHAM group (*P* < 0.05). Compared with the MR group, the normal-dose rosuvastatin therapy group showed significantly higher levels of claudin-5, VE-cadherin, and occludin (*P* < 0.05), whereas the high-dose rosuvastatin therapy group showed significant increases in VE-cadherin and occludin and no significant difference in claudin-5 (*P* < 0.05) (**Figures 3H–J**).

Thus, the assessment of the effects of normal-dose and high-dose rosuvastatin therapy on BBB integrity indicated that the rosuvastatin therapy provided neurovascular protection by increasing the levels of TJs and AJs to protect against rt-PA reperfusion after MCAO.

### Rosuvastatin Reduced MMP Expression and Increased TIMP Expression in Peri-Infarct Regions

**FIGURE 4:**
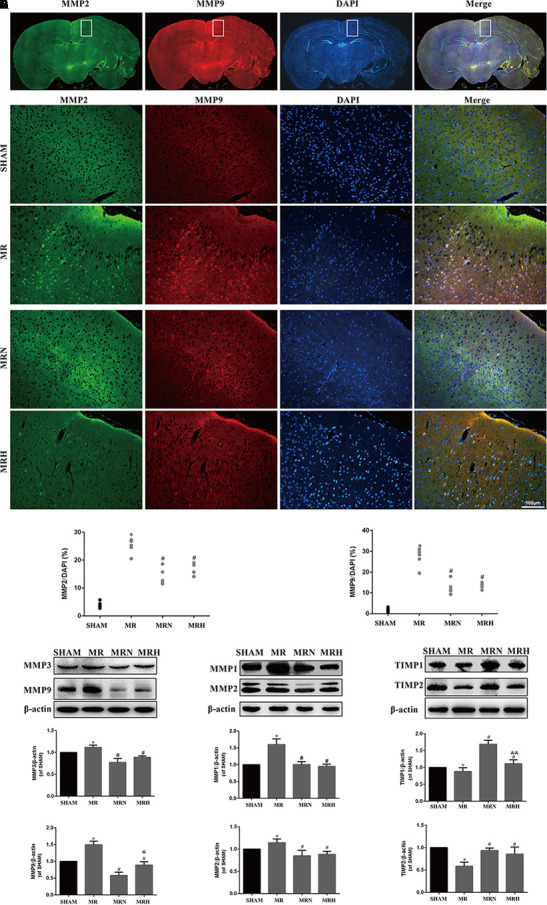
Rosuvastatin reduced MMP expression and increased TIMPs expression in peri-infarct regions. **(A)** Representative images show MMP-2 (green) and MMP-9 (red) in the MR group; DAPI was used to stain the cell nuclei. The white box magnified in **(B)** shows the peri-infarct regions. The magnified images show MMP-2 and MMP-9 staining in the peri-infarct regions in SHAM, MR, MRN, and MRH groups (scale bar = 100 μm). **(C,D)** The MMP-2-labeled particle numbers (green)/DAPI (blue) and MMP-9-labeled particle numbers (red)/DAPI (blue) are presented as the means + SEM ^∗^*P* < 0.05 compared to the SHAM group, ^#^*P* < 0.05 compared to MR group, *n* = 6. **(E–G)** Immunoblots were used to test MMP-3, MMP-9, MMP-1, MMP-2, TIMP-1, and TIMP-2 levels in the peri-infarct regions. Similar results from six independent experiments are shown by representative blots and statistical charts. Data are presented as the means + SEM and were analyzed by one-way ANOVA. ^∗^*P* < 0.05 compared to SHAM, ^#^*P* < 0.05 compared to MR, ^&^*P* < 0.05, ^&&^*P* < 0.01 compared to MRN.

Previous studies have shown that rt-PA reperfusion significantly increases MMP-3 and MMP-9 levels, leading to the degradation of the basal lamina and TJ proteins essential for the barrier function of neurovascular units (Kumari et al., 2011). MMP-1 and MMP-2 are also capable of degrading claudin and occludin but not Zo-1, all key components of the BBB (Wu et al., 2015). Herein, MMP-2 (green) and MMP-9 (red) (**Figures 4A,B**) exhibited critical roles in the disruption of the BBB that occurred during AIS as both were upregulated in peri-infarct regions, as assessed by IF detection (**Figures 4C,D**); however, the increases in MMP-2 and MMP-9 were reduced following both normal- and high-dose rosuvastatin therapy (**Figure 4B**, *P* < 0.05 vs. SHAM). Consistent with the IHC results, MMP-1, MMP-2, MMP-3, and MMP-9 activity assessed by Western blot were much greater in the MR group than in the SHAM group, whereas pretreatment with both normal and high doses of rosuvastatin significantly inhibited the secretion of MMP-1, 2, 3, and 9 (**Figures 4E,F**, *P* < 0.05 vs. SHAM).

Conversely, TIMP-1 and TIMP-2 are crucial endogenous inhibitors of MMPs. An imbalance between MMPs and TIMPs could induce disruptions in the BBB, contributing to cerebral edema and BBB damage. Both normal and high doses of rosuvastatin reversed the rt-PA-reperfusion-induced TIMP-1 and TIMP-2 degradation seen in peri-infarct tissues (**Figure 4G**). Notably, of the rosuvastatin-pretreatment groups, the normal-dose therapy group exhibited greater upregulation of TIMP-1 and TIMP-2 activity than did the MRH group (*P* < 0.05).

### The Rosuvastatin-Mediated Reduction in the BBB Damage Induced by rt-PA Reperfusion After MCAO Was Associated With the Expression of PDGFR-α and LRP-1

**FIGURE 5:**
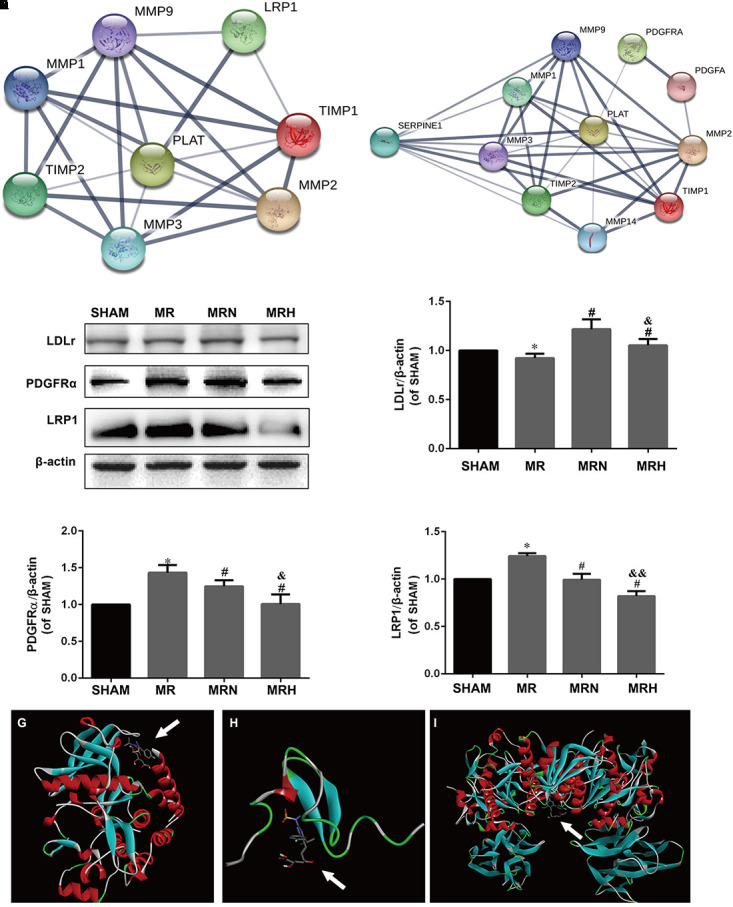
Rosuvastatin reductions in the BBB damage induced by rt-PA-reperfusion after MCAO are associated with the expression of PDGFR-α, LRP-1, and LDLr. **(A,B)** The interaction networks between tPA, LRP1, PDGFR-α, TIMP-1, TIMP-2, MMP-1, MMP-2, MMP-3, and MMP-9 were searched using the STRING database. PPI scores are shown in **Supplementary Table S1**. **(C)** The protein bands for PDGFR-α, LRP-1, LDLr, and β-actin detected using Western blotting and exposed by a Tanon 2500/2500R. **(D–F)** The relative densities were assessed by calculating the ratios of PDGFR-α, LRP-1, and LDLr proteins to β-actin and comparing the differences with the SHAM group (^∗^*P* < 0.05), among the MRN, MRH, and MR groups (^#^*P* < 0.05), and between the MRN and MRH groups (^&^*P* < 0.05, ^&&^*P* < 0.01). Means ± SEM, *n* = 6. **(G)** The molecular docking of rosuvastatin to PDGFR-α is visualized. **(H)** The molecular docking of rosuvastatin to LRP-1 is visualized. **(I)** The molecular docking of rosuvastatin to LDLr is visualized. The affinity was calculated and is shown in **Supplementary Table S2**.

Several studies have revealed that rt-PA increases MMP-2 expression via astrocyte PDGFR-α stimulation and upregulates MMP-1, MMP-3, and MMP-9 activity by selective LRP1 activation in ECs to disrupt BBB integrity. To pinpoint the potential interactions between rt-PA and binding proteins, an interplay network was first constructed using the STRING database for LRP1, PDGFR-α and the associated MMP and TIMP proteins. Although LDLr and LRP1, both members of the LDLr-related receptor family, can be used to transport different molecules, including stress factors and antistress factors, LDLr was not searched for in the database. As shown in **Figures 5A,B** and **Supplementary Table S1**, the correlation between LRP1 and tPA (PLAT, plasminogen activator, tissue) (protein–protein interaction (PPI) score = 0.978) was stronger than the correlations between other protein pairs, suggesting that LRP1 might be the easiest protein for other proteins to combine with. Then, Western blotting indicated that the protein expression levels of LRP1 and PDGFR-α were increased in the mice that received rt-PA reperfusion after MCAO injury; however, the expression of PDGFR-α (**Figures 5C,E**) and LRP1 (**Figures 5C,F**) were significantly lower in the MRN and MRH groups (*P* < 0.05). These findings indicate that rosuvastatin may compete with rt-PA and hinder the binding between rt-PA and LRP1. In this study, we found that in contrast to the levels of LRP1, the levels of LDLr were downregulated in the MR group but upregulated in the normal-dose rosuvastatin therapy group; this upregulation was not seen in the high-dose rosuvastatin therapy group (**Figures 5C,D**). To explore possible interactions between rosuvastatin and the PDGFR-α, LRP-1 and LDLr proteins, molecular docking analysis (**Figures 5G–I**) was performed to calculate affinity (Δ*G* = binding energies). As shown in **Supplementary Table S2**, rosuvastatin bound to PDGFR-α with a low binding energy of -7.9, to LRP1 with a binding energy of -6.7, and to LDLr with a binding energy of -8.2. These results suggested that for the affinity of rosuvastatin for PDGFR-α, LRP1, and LDLr *in vivo* when Δ*G* was negative, the smaller the value, the stronger the affinity. Similarly, K*i* can be used to explain the activities of ligands toward receptors, including PDGFR-α (1758 nmol/l), LRP1 (13156 nmol/l), and LDLr (1063 nmol/l); these values suggest a small number of receptors bound to rosuvastatin.

### The Rosuvastatin-Mediated Reduction in BBB Damage Induced by rt-PA Reperfusion After MCAO Was Associated With MAPK Pathways

**FIGURE 6:**
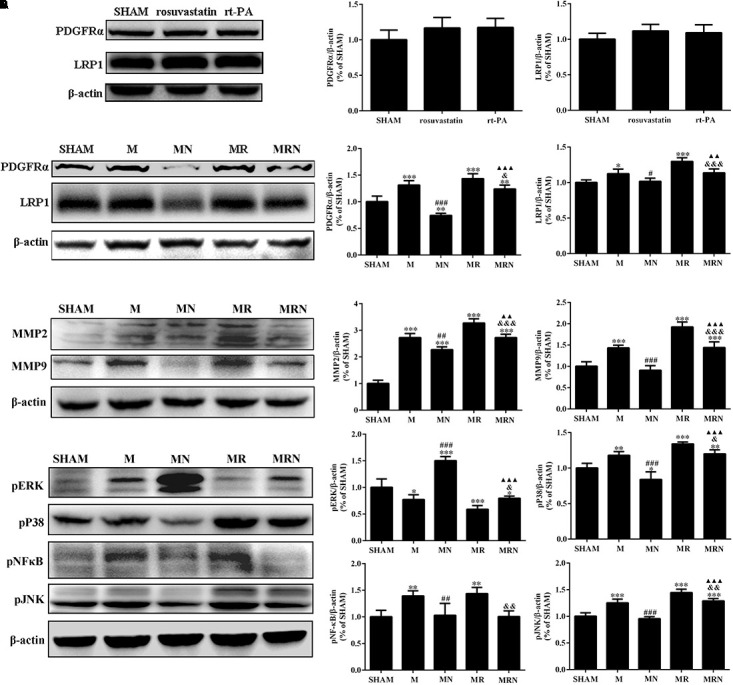
The rosuvastatin-mediated reduction in BBB damage induced by rt-PA reperfusion after MCAO was associated with MAPK pathways. **(A)** The bands for the PDGFR-α, LRP-1, and β-actin proteins in the SHAM, 1 mg/kg rosuvastatin-alone treatment (rosuvastatin), and rt-PA-alone treatment (rt-PA) groups determined by Western blotting. The relative densities were assessed by calculating the ratios of the PDGFR-α and LRP-1 proteins to β-actin and comparing the differences with the SHAM group, *P* > 0.05. Means ± SEM, *n* = 3. **(B)** The bands for the PDGFR-α, LRP-1, and β-actin proteins in the SHAM group, mice that underwent MCAO for 3 h followed by reperfusion for 24 h (M), mice receiving a high dose of rosuvastatin prior to MCAO for 3 h followed by reperfusion for 24 h (MN), mice that underwent MCAO for 3 h followed by rt-PA reperfusion for 24 h (MR), and mice pretreated with a high dose of rosuvastatin prior to MCAO for 3 h followed by rt-PA reperfusion for 24 h (MRN) were determined by Western blotting. The relative densities were assessed by calculating the ratios of the PDGFR-α and LRP-1 proteins to β-actin and comparing the differences with the SHAM group (^∗^*P* < 0.05, ^∗∗^*P* < 0.01, ^∗∗∗^*P* < 0.001), the M group (^#^*P* < 0.05, ^###^*P* < 0.001), the MR group (^&^*P* < 0.05, ^&&&^*P* < 0.001), and the MN group (*P* < 0.01, *P* < 0.001). Means ± SEM, *n* = 3. **(C)** The bands for the MMP2, MMP9, and β-actin proteins were detected by Western blotting of samples from the SHAM, M, MN, MR, and MRN groups. The relative densities were assessed by calculating the ratios of the MMP2 and MMP9 proteins to β-actin and comparing the differences with the SHAM group (^∗∗∗^*P* < 0.001), the M group (^##^*P* < 0.01, ^###^*P* < 0.001), the MR group (^&&&^*P* < 0.001), and the MN group (*P* < 0.01 and *P* < 0.001). Means ± SEM, *n* = 3. **(D)** The bands for the pERK, pP38, pJNK, pNFκB, and β-actin proteins were detected by Western blotting samples from the SHAM, M, MN, MR, and MRN groups. The relative densities were assessed by calculating the ratios of pERK, pP38, pJNK, and pNFκB proteins to β-actin and comparing the differences with the SHAM group (^∗^*P* < 0.05, ^∗∗^*P* < 0.01, ^∗∗∗^*P* < 0.001), the M group (^##^*P* < 0.01, ^###^*P* < 0.001), the MR group (^&^*P* < 0.05, ^&&^*P* < 0.01), and the MN group (*P* < 0.001). Means ± SEM, *n* = 3.

Following ischemic stroke, a significant increase in tissue plasminogen activator-dependent cerebrovascular permeability occurs via signaling through the activated PDGFRα pathway (Su et al., 2017) and LRP1 pathway (Zhang et al., 2009). Although significant differences were not observed among the SHAM, rosuvastatin-alone, and rt-PA-alone groups (**Figure 6A**), rt-PA reperfusion after MCAO induced a substantial increase in the expression of PDGFR-α (*P* < 0.001), LRP1 (*P* < 0.05), MMP2 (*P* < 0.001), and MMP9 (*P* < 0.001) compared to that from MCAO alone, and this effect was significantly attenuated in the mice pretreated with rosuvastatin (**Figures 6B,C**, *P* < 0.05). We also evaluated the upstream pP38, pJNK, and pERK, and pNF-κB pathways; the levels of these intermediates were significantly increased in the MCAO (M) and MR groups, and this effect was significantly reversed in the mice pretreated with rosuvastatin (**Figure 6D**, *P* < 0.05). Receptor levels were further assessed to determine the possible inhibitory effect of rosuvastatin on the deleterious effects of rt-PA, and receptor levels were increased in the MRN group (*P* < 0.05) compared to those of the mice in the MR group; this finding was similar in terms of MMPs. The quantitative data showing the relative levels of pP38 and pJNK, but not pNF-κB, are presented in **Figure 6D**. Thus, PDGFRα and LRP-1 were absolutely required for tPA-mediated MMP induction.

## Discussion

A much higher dose of tPA was used in the present study than that used in the clinic because an approximately 10-fold difference in the fibrin-specific enzyme activity of human recombinant tPA has been observed in humans and rodents. The 10-fold higher dose of rt-PA used here and the dose used in clin permeability. Importantly, a reduction in permeability will promote a decrease in hemorrhaging. Here, we hypothesized that a single statin treatment would decrease BBB permeability and subsequently prevent HT, as the administration of a single statin treatment (atorvastatin and simvastatin) 1 h prior sepsis induction reversed microvascular dysfunction in septic subjects in a previous study (Reis et al., 2017). Based on our findings, rosuvastatin played a profound and previously undiscovered role in regulating BBB permeability following rt-PA reperfusion-induced HT in mice. Both normal and high doses of rosuvastatin nearly abolished the ischemia-induced BBB permeability.

According to several studies, rt-PA increases the levels of constitutively expressed MMPs, including MMP-9, MMP-2, and MMP-3. Indeed, MMP-1 and MMP-2 appear to initiate the damage cascade early during the acute rt-PA thrombolysis phase within 2–6 h (median 3.8 h) after ischemic brain injury, and periods of enhanced BBB permeability are present at 4–8 h and again at 12–16 h after stroke onset. Inducible MMPs, such as MMP-9 and MMP-3, perpetuate the white matter and BBB damage over hours and days (Yang and Rosenberg, 2015); although the highest levels were observed at 24 h, the disruption of the BBB can persist for weeks (Khatri et al., 2012). These factors are involved in the activation of MMPs that are directly responsible for the disruptions in the TJs, AJs and the digestion of collagen type IV in the endothelium, thereby facilitating blood cell extravasation and vasogenic edema from BBB (Hernandes et al., 2018). For BBBs with weak basal permeability levels, thrombolysis therapy causes intracellular Ca^2+^ overload, inflammation and oxidative stress followed by postperfusion hemorrhaging (Thompson and Ronaldson, 2014). Hemorrhaging and vasogenic edema induce neuron damage and expanded brain ischemia to trigger neural deficiencies, such as a brain hernia, ultimately causing death.

Consistent with the results from clinical trials (Zhang et al., 2009), neurological deficits were unchanged in subjects pretreated with rosuvastatin, while the hemorrhagic zones were smaller after rosuvastatin therapy, as evidenced by the reduction in Perls’ iron and Evans blue leakage from the BBB. Meanwhile, the hemorrhagic content was decreased with rosuvastatin therapy, as evidenced by the decreased levels of Evans blue dye, hemoglobin and biotin leakage. The reduction in HT inhibited the mortality induced by the rosuvastatin pretreatment, but this change was not significantly different between the mice receiving a normal dose and those receiving a high dose of rosuvastatin. The protective effects of rosuvastatin were closely related to the attenuation of BBB damage-associated MMP activation that triggers hemorrhages in subjects with ischemic strokes following thrombolysis therapy. Both normal-dose and high-dose rosuvastatin therapy abolished the initial effects on MMP-1 and MMP-2. After MMP activation, TIMPs bind to the active site and block substrate availability, thus inhibiting MMP-9; TIMP-2 appears to inhibit MMP-2 (Benjamin and Khalil, 2012). This increase in TIMP-1 levels resulted in a greater inhibition or attenuation of MMP-9 actions in the normal-dose rosuvastatin-treated mice than in the high-dose rosuvastatin-treated mice.

Previous data have revealed that increased MMP-2, MMP-1, MMP-3, and MMP-9 expression levels might occur via PDGFR-α (Ma et al., 2011) and LRP1 stimulation of the BBB (Cheon et al., 2017; Niego et al., 2017). In this study, PPI networks among rt-PA, which is called tissue plasminogen activator (PLAT, tPA) *in vivo*, and PDGFR-α, LRP1, MMP-2, MMP-1, MMP-3, and MMP-9 were constructed based on the STRING database; this analysis suggested a strong interaction between tPA and LRP1, which showed the highest confidence score. Due to the different algorithms for the binding of the enzyme rt-PA to receptors and the binding of the drug rosuvastatin to proteins, binding forces were identified by Western blotting. To further assess the competitive relationship between tPA and rosuvastatin binding of PDGFR-α and LRP1, Western blotting analysis was performed, which showed that both normal and high doses of rosuvastatin inhibited the expression of PDGFR-α and LRP1 in a dose-dependent manner. Therefore, these results explain why rosuvastatin therapy likely contributed to a reduction in the effects induced by rt-PA reperfusion after MCAO on MMPs and TIMPs by decreasing the levels of PDGFR-α and LRP1. Several notable exceptions have been identified, such as the effects of normal-dose rosuvastatin therapy on the levels of TIMP-1, MMP-9, claudin-5, and LDLr. The consistent expression of LDLr along with changes in TIMP-1 and MMP-9 actions suggests that the upregulation of TIMP-1 and the downregulation of MMP-9 induced by a normal dose of rosuvastatin were related to an upregulation of LDLr. In the normal-dose rosuvastatin therapy mice, the improvements in TJs and AJs in the BBB damaged by MMPs were greater than those in the high-dose rosuvastatin therapy mice.

As shown in previous studies, tPA functions as a cytokine that binds to the cell membrane receptors LRP-1 and PDGFRα and triggers the phosphorylation of JNK, P38, NF-κB, thereby inducing the expression of the MMP2 and MMP9 genes (Hu et al., 2006; Ma et al., 2011; Cheon et al., 2017; Su et al., 2017). We further investigated the mechanisms and showed that LRP1 and PDGFRα were not activated in mice that received high-dose rosuvastatin treatment alone and rt-PA treatment alone but were activated in the mice receiving MCAO followed by rt-PA reperfusion for 24 h and MCAO alone. The interactions between tPA and LRP1 or PDGFRα during cerebral ischemia increase the expression of MMP2 and MMP9. The increased permeability of BBB mediated by MMP2 and MMP9, the reduction in P38 and JNK levels, and the increase in ERK-mediated signaling in endothelial cells were reversed by rosuvastatin. The results established that the mechanism by which rosuvastatin blocked the deleterious effects of rt-PA was to inhibit PDGFRα and LRP1 signaling and the subsequent activation of P38 and JNK, as well as to promote ERK phosphorylation.

This study has some limitations. Further studies using knockdown experiments will be necessary to identify the exact targets of LRP1, LDLr, and PDGFR-α in the brain. Finally, whether rosuvastatin is a target for the modulation of BBB permeability for rt-PA therapies beyond the current clinical time window should be further studied to reduce the complications of hemorrhages, such as headaches, seizures, and brain herniations.

## Conclusion

Both normal and high doses of rosuvastatin exert beneficial effects on preventing rt-PA-associated hemorrhages in mice with brain ischemia to reduce mortality but did not alter cerebral blood reflow or neural function. This prevention was closely associated with the reduction of BBB damage by inhibiting MMPs and activating TIMPs through LRP1 and PDGFR-α. These benefits might be associated with the inactivation of pJNK and pP38 and the activation of pERK, further inhibiting the expression of MMPs and activating TIMP-1.

## Author Contributions

Y-SZ conceived and designed the experiments. DL, H-CM, Y-BL, and B-DX performed the experiments. DL and H-CM analyzed the data. DL, H-CM, A-DX, and Y-SZ prepared the manuscript.

## Conflict of Interest Statement

The authors declare that the research was conducted in the absence of any commercial or financial relationships that could be construed as a potential conflict of interest.

## Footnotes

1
https://www.ncbi.nlm.nih.gov/

2
http://www.rcsb.org/

## Abbreviations

AIS: acute ischemic stroke
BBB: blood–brain barrier
HMG-CoA: 3-hydroxy-methylglutaryl coenzyme A
HT: hemorrhage transformation
LRP1: low-density lipoprotein receptor-related protein 1
MCAO, or M: middle cerebral artery occlusion
MH: MCAO mice pretreated with a high dose of rosuvastatin
MMPs: matrix metalloproteinases
MR: MCAO mice treated with rt-PA
MRH: MCAO mice treated with rt-PA were pretreated with a high dose of rosuvastatin
MRN: MCAO mice treated with rt-PA were pretreated with a normal dose of rosuvastatin
PDGFR-α: platelet-derived growth factor receptor α
pERK: phosphorylated extracellular regulated protein kinase
pJNK: phosphorylated c-jun-N-terminal kinase
pP38: phosphorylated mitogen-activated protein kinase P38
rt-PA: recombinant tissue plasminogen activator
sICHs: symptomatic intracranial hemorrhages
TIMPs: tissue inhibitor of metalloproteinases
TJ: tight junction.

## References

[B1] Benjamin M. M. Khalil R. A. (2012). Matrix metalloproteinase inhibitors as investigative tools in the pathogenesis and management of vascular disease. *EXS* 103 209–279. 10.1007/978-3-0348-0364-9_7 22642194 PMC3367802

[B2] Cai H. Ma Y. Jiang L. Mu Z. Jiang Z. Chen X. (2017). Hypoxia response element-regulated MMP-9 promotes neurological recovery via glial scar degradation and angiogenesis in delayed stroke. *Mol. Ther.* 25 1448–1459. 10.1016/j.ymthe.2017.03.020 28396199 PMC5474960

[B3] Cappellari M. Bovi P. Moretto G. Zini A. Nencini P. Sessa M. (2013). The THRombolysis and STatins (THRaST) study. *Neurology* 80 655–661. 10.1212/WNL.0b013e318281cc83 23345634 PMC3590058

[B4] Chang J. Mancuso M. R. Maier C. Liang X. Yuki K. Yang L. (2017). Gpr124 is essential for blood-brain barrier integrity in central nervous system disease. *Nat. Med.* 23 450–460. 10.1038/nm.4309 28288111 PMC5559385

[B5] Cheon S. Y. Kim S. Y. Kam E. H. Lee J. H. Kim J. M. Kim E. J. (2017). Isoflurane preconditioning inhibits the effects of tissue-type plasminogen activator on brain endothelial cell in an in vitro model of ischemic stroke. *Int. J. Med. Sci.* 14 425–433. 10.7150/ijms.18037 28539818 PMC5441034

[B6] Emberson J. Lees K. R. Lyden P. Blackwell L. Albers G. Bluhmki E. (2014). Thrombolysis trialists’ collaborative, effect of treatment delay, age, and stroke severity on the effects of intravenous thrombolysis with alteplase for acute ischaemic stroke: a meta-analysis of individual patient data from randomised trials. *Lancet* 384 1929–1935. 10.1016/S0140-6736(14)60584-5 25106063 PMC4441266

[B7] Erdur H. Polymeris A. Grittner U. Scheitz J. F. Tutuncu S. Seiffge D. J. (2018). A score for risk of thrombolysis-associated hemorrhage including pretreatment with statins. *Front. Neurol.* 9:74. 10.3389/fneur.2018.00074 29503629 PMC5820302

[B8] Fan X. Jiang Y. Yu Z. Yuan J. Sun X. Xiang S. (2014). Combination approaches to attenuate hemorrhagic transformation after tPA thrombolytic therapy in patients with poststroke hyperglycemia/diabetes. *Adv. Pharmacol.* 71 391–410. 10.1016/bs.apha.2014.06.007 25307224

[B9] Goldstein L. B. Amarenco P. Szarek M. Callahan A. III Hennerici M. Sillesen H. (2008). Hemorrhagic stroke in the stroke prevention by aggressive reduction in cholesterol levels study. *Neurology* 70 2364–2370. 10.1212/01.wnl.0000296277.63350.77 18077795

[B10] Goldstein L. B. Amarenco P. Zivin J. Messig M. Altafullah I. Callahan A. (2009). Prevention by aggressive reduction in cholesterol levels, statin treatment and stroke outcome in the stroke prevention by aggressive reduction in cholesterol levels (SPARCL) trial. *Stroke* 40 3526–3531. 10.1161/STROKEAHA.109.557330 19745172

[B11] Goyal M. Menon B. K. van Zwam W. H. Dippel D. W. Mitchell P. J. Demchuk A. M. (2016). Endovascular thrombectomy after large-vessel ischaemic stroke: a meta-analysis of individual patient data from five randomised trials. *Lancet* 387 1723–1731. 10.1016/S0140-6736(16)00163-X 26898852

[B12] Hernandes M. S. Lassegue B. Hilenski L. L. Adams J. Gao N. Kuan C. Y. (2018). Polymerase delta-interacting protein 2 deficiency protects against blood-brain barrier permeability in the ischemic brain. *J. Neuroinflammation* 15:45. 10.1186/s12974-017-1032-1 29452577 PMC5816395

[B13] Hong C. Schuffler A. Kauhl U. Cao J. Wu C. F. Opatz T. (2017). Identification of NF-kappaB as determinant of posttraumatic stress disorder and its inhibition by the Chinese herbal remedy free and easy wanderer. *Front. Pharmacol.* 8:181. 10.3389/fphar.2017.00181 28428751 PMC5382210

[B14] Hu K. Yang J. Tanaka S. Gonias S. L. Mars W. M. Liu Y. (2006). Tissue-type plasminogen activator acts as a cytokine that triggers intracellular signal transduction and induces matrix metalloproteinase-9 gene expression. *J. Biol. Chem.* 281 2120–2127. 10.1074/jbc.M504988200 16303771

[B15] Jia L. Chopp M. Zhang L. Lu M. Zhang Z. (2010). Erythropoietin in combination of tissue plasminogen activator exacerbates brain hemorrhage when treatment is initiated 6 hours after stroke. *Stroke* 41 2071–2076. 10.1161/STROKEAHA.110.586198 20671252 PMC2950698

[B16] Jickling G. C. Liu D. Stamova B. Ander B. P. Zhan X. Lu A. (2014). Hemorrhagic transformation after ischemic stroke in animals and humans. *J. Cereb. Blood Flow Metab.* 34 185–199. 10.1038/jcbfm.2013.203 24281743 PMC3915212

[B17] Jones P. H. Davidson M. H. Stein E. A. Bays H. E. McKenney J. M. Miller E. (2003). Comparison of the efficacy and safety of rosuvastatin versus atorvastatin, simvastatin, and pravastatin across doses (STELLAR^∗^ Trial). *Am. J. Cardiol.* 92 152–160. 10.1016/S0002-9149(03)00530-7 12860216

[B18] Khan M. Sakakima H. Dhammu T. S. Shunmugavel A. Im Y. B. Gilg A. G. (2011). S-nitrosoglutathione reduces oxidative injury and promotes mechanisms of neurorepair following traumatic brain injury in rats. *J. Neuroinflammation* 8:78. 10.1186/1742-2094-8-78 21733162 PMC3158546

[B19] Khatri R. McKinney A. M. Swenson B. Janardhan V. (2012). Blood-brain barrier, reperfusion injury, and hemorrhagic transformation in acute ischemic stroke. *Neurology* 79 S52–S57. 10.1212/WNL.0b013e3182697e70 23008413

[B20] Knecht T. Story J. Liu J. Davis W. Borlongan C. V. Dela Pena I. C. (2017). Adjunctive therapy approaches for ischemic stroke: innovations to expand time window of treatment. *Int. J. Mol. Sci.* 18:E2756. 10.3390/ijms18122756 29257093 PMC5751355

[B21] Kumari R. Willing L. B. Patel S. D. Baskerville K. A. Simpson I. A. (2011). Increased cerebral matrix metalloprotease-9 activity is associated with compromised recovery in the diabetic db/db mouse following a stroke. *J. Neurochem.* 119 1029–1040. 10.1111/j.1471-4159.2011.07487.x 21923664 PMC3217107

[B22] Lenglet S. Montecucco F. Denes A. Coutts G. Pinteaux E. Mach F. (2014). Recombinant tissue plasminogen activator enhances microglial cell recruitment after stroke in mice. *J. Cereb. Blood Flow Metab.* 34 802–812. 10.1038/jcbfm.2014.9 24473480 PMC4013777

[B23] Lu D. Liu Y. Mai H. Zang J. Shen L. Zhang Y. (2018). Rosuvastatin reduces neuroinflammation in the hemorrhagic transformation after rt-PA treatment in a mouse model of experimental stroke. *Front. Cell. Neurosci.* 12:225. 10.3389/fncel.2018.00225 PMC6082938 30116175

[B24] Ma Q. Huang B. Khatibi N. Rolland W. II Suzuki H. Zhang J. H. (2011). PDGFR-alpha inhibition preserves blood-brain barrier after intracerebral hemorrhage. *Ann. Neurol.* 70 920–931. 10.1002/ana.22549 22190365 PMC3405848

[B25] Nakamura T. Keep R. F. Hua Y. Schallert T. Hoff J. T. Xi G. (2003). Deferoxamine-induced attenuation of brain edema and neurological deficits in a rat model of intracerebral hemorrhage. *Neurosurg. Focus* 15:ECP4. 10.3171/foc.2003.15.4.10 15344903

[B26] National Institute of Neurological Disorders and Stroke rt-PA Stroke Study Group (1995). Tissue plasminogen activator for acute ischemic stroke. *N. Engl. J. Med.* 333 1581–1587. 7477192 10.1056/NEJM199512143332401

[B27] Niego B. Broughton B. R. S. Ho H. Sobey C. G. Medcalf R. L. (2017). LDL receptor blockade reduces mortality in a mouse model of ischaemic stroke without improving tissue-type plasminogen activator-induced brain haemorrhage: towards pre-clinical simulation of symptomatic ICH. *Fluids Barriers CNS* 14:33. 10.1186/s12987-017-0081-2 29157263 PMC5696777

[B28] Peng S. X. Rockafellow B. A. Skedzielewski T. M. Huebert N. D. Hageman W. (2009). Improved pharmacokinetic and bioavailability support of drug discovery using serial blood sampling in mice. *J. Pharm. Sci.* 98 1877–1884. 10.1002/jps.21533 18803263

[B29] Pfeilschifter W. Spitzer D. Czech-Zechmeister B. Steinmetz H. Foerch C. (2011). Increased risk of hemorrhagic transformation in ischemic stroke occurring during warfarin anticoagulation: an experimental study in mice. *Stroke* 42 1116–1121. 10.1161/STROKEAHA.110.604652 21330626

[B30] Reis P. A. Alexandre P. C. B. D’Avila J. C. Siqueira L. D. Antunes B. Estato V. (2017). Statins prevent cognitive impairment after sepsis by reverting neuroinflammation, and microcirculatory/endothelial dysfunction. *Brain Behav. Immun.* 60 293–303. 10.1016/j.bbi.2016.11.006 27833044

[B31] Saver J. L. Goyal M. Bonafe A. Diener H. C. Levy E. I. Pereira V. M. (2015). Investigators, stent-retriever thrombectomy after intravenous t-PA vs. t-PA alone in stroke. *N. Engl. J. Med.* 372 2285–2295. 10.1056/NEJMoa1415061 25882376

[B32] Simao F. Ustunkaya T. Clermont A. C. Feener E. P. (2017). Plasma kallikrein mediates brain hemorrhage and edema caused by tissue plasminogen activator therapy in mice after stroke. *Blood* 129 2280–2290. 10.1182/blood-2016-09-740670 28130211 PMC5399481

[B33] Su E. J. Cao C. Fredriksson L. Nilsson I. Stefanitsch C. Stevenson T. K. (2017). Microglial-mediated PDGF-CC activation increases cerebrovascular permeability during ischemic stroke. *Acta Neuropathol.* 134 585–604. 10.1007/s00401-017-1749-z 28725968 PMC5587628

[B34] Suzuki Y. Nagai N. Umemura K. (2011). Novel situations of endothelial injury in stroke–mechanisms of stroke and strategy of drug development: intracranial bleeding associated with the treatment of ischemic stroke: thrombolytic treatment of ischemia-affected endothelial cells with tissue-type plasminogen activator. *J. Pharmacol. Sci.* 116 25–29. 10.1254/jphs.10R27FM 21498957

[B35] Szklarczyk D. Morris J. H. Cook H. Kuhn M. Wyder S. Simonovic M. (2017). The STRING database in 2017: quality-controlled protein-protein association networks, made broadly accessible. *Nucleic Acids Res.* 45 D362–D368. 10.1093/nar/gkw937 27924014 PMC5210637

[B36] Tejima E. Katayama Y. Suzuki Y. Kano T. Lo E. H. (2001). Hemorrhagic transformation after fibrinolysis with tissue plasminogen activator: evaluation of role of hypertension with rat thromboembolic stroke model. *Stroke* 32 1336–1340. 10.1161/01.STR.32.6.1336 11387496

[B37] Thompson B. J. Ronaldson P. T. (2014). Drug delivery to the ischemic brain. *Adv. Pharmacol.* 71 165–202. 10.1016/bs.apha.2014.06.013 25307217 PMC4281266

[B38] Turner R. J. Sharp F. R. (2016). Implications of MMP9 for blood brain barrier disruption and hemorrhagic transformation following ischemic stroke. *Front. Cell. Neurosci.* 10:56. 10.3389/fncel.2016.00056 26973468 PMC4777722

[B39] Wu K. Fukuda K. Xing F. Zhang Y. Sharma S. Liu Y. (2015). Roles of the cyclooxygenase 2 matrix metalloproteinase 1 pathway in brain metastasis of breast cancer. *J. Biol. Chem.* 290 9842–9854. 10.1074/jbc.M114.602185 25691572 PMC4392281

[B40] Yaghi S. Willey J. Z. Cucchiara B. Goldstein J. N. Gonzales N. R. Khatri P. (2017). Treatment and outcome of hemorrhagic transformation after intravenous alteplase in acute ischemic stroke: a scientific statement for healthcare professionals from the American Heart Association/American Stroke Association. *Stroke* 48 e343–e361. 10.1161/STR.0000000000000152 29097489

[B41] Yang Y. Rosenberg G. A. (2015). Matrix metalloproteinases as therapeutic targets for stroke. *Brain Res.* 1623 30–38. 10.1016/j.brainres.2015.04.024 25916577 PMC4569515

[B42] Zhang C. An J. Strickland D. K. Yepes M. (2009). The low-density lipoprotein receptor-related protein 1 mediates tissue-type plasminogen activator-induced microglial activation in the ischemic brain. *Am. J. Pathol.* 174 586–594. 10.2353/ajpath.2009.080661 19147818 PMC2630566

