# Correction: Beneficial role of rosuvastatin in blood–brain barrier damage following experimental ischemic stroke

**DOI:** 10.3389/fphar.2026.1895293

**Published:** 2026-06-26

**Authors:** Dan Lu, Hong-Cheng Mai, Yu-Bin Liang, Bing-Dong Xu, An-Ding Xu, Yu-Sheng Zhang

**Affiliations:** 1 Department of Neurology and Stroke Center, The First Affiliated Hospital, Jinan University, Guangzhou, China; 2 Clinical Neuroscience Institute of Jinan University, Jinan University, Guangzhou, China

**Keywords:** tissue type plasminogen activator therapy, blood–brain barrier, rosuvastatin, tight junction protein, matrix metalloproteinase, focal cerebral ischemia

In the published article, there was a mistake in Figure 2G as published. The representative Perls’ iron staining image panel was inadvertently assembled incorrectly during figure preparation. In the corrected replacement figure submitted with this correction, the corresponding Perls’ iron staining images are shown as panels E–H. The corrected Figure 2 appears below.

**FIGURE 2:**
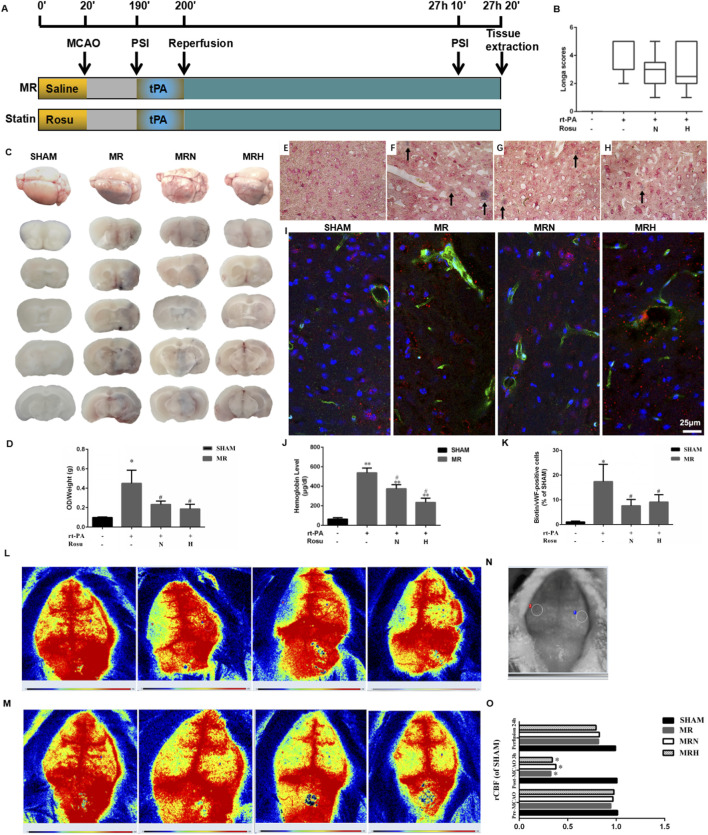
Rosuvastatin decreased BBB permeability at 24 h following rt-PA reperfusion after brain ischemia but did not alter cerebral blood reflow. **(A)** Experimental timeline showing MCAO, rosuvastatin/saline administration, rt-PA reperfusion, laser speckle imaging, and tissue collection. **(B)** Neurological deficits were evaluated in a blinded manner by determining the Longa score before MCAO and at 24 h after reperfusion in the SHAM, MR, MRN, and MRH groups. **(C)** Representative whole-brain and coronal brain sections from the SHAM, MR, MRN, and MRH groups after MCAO followed by 24 h reperfusion. The dark blue color indicates Evans blue-stained leakage areas. **(D)** Evans blue leakage in the brain was quantified spectrophotometrically and normalized to brain tissue weight. **(E–H)** Representative Perls’ iron staining images showing microvascular hemorrhage in the SHAM, MR, MRN, and MRH groups, respectively. The dark blue areas indicated by black arrows are associated with hemorrhage. Scale bar, 50 μm. **(I)** Representative images showing the Sulfo-NHS-Biotin tracer extravasation assay. Biotin leakage is shown in green, and vWF-positive vessels are shown in red. Scale bar, 25 μm. **(J)** Quantitative analysis of intracerebral hemorrhage using a hemoglobin assay. **(K)** Quantitative analysis of biotin/vWF-positive cells normalized to the SHAM group. **(L)** Representative cerebral blood flow images before reperfusion in the SHAM, MR, MRN, and MRH groups captured by 2-D laser speckle imaging. **(M)** Representative cerebral blood flow images after 24 h reperfusion in the SHAM, MR, MRN, and MRH groups captured by 2-D laser speckle imaging. **(N)** Circles indicate the monitored areas for cerebral blood flow analysis. **(O)** Quantitative analysis of relative cerebral blood flow. Data are presented as mean ± SEM. *P < 0.05 and **P < 0.01 compared with SHAM; #P < 0.05 compared with MR; &P < 0.05 compared with MRN. MR, mice that underwent MCAO for 3 h followed by rt-PA reperfusion for 24 h; MRN, mice pretreated with a normal dose of rosuvastatin (1 mg/kg) prior to MCAO for 3 h followed by rt-PA reperfusion for 24 h; MRH, mice pretreated with a high dose of rosuvastatin (5 mg/kg) prior to MCAO for 3 h followed by rt-PA reperfusion for 24 h.

The original article has been updated.

