# Supplementary material for: Beneficial Role of Rosuvastatin in Blood–Brain Barrier Damage Following Experimental Ischemic Stroke
Source: Front Pharmacol. 2018 Aug 21;9:926. doi: 10.3389/fphar.2018.00926 (PMC6110873; doi:10.3389/fphar.2018.00926)
Supplement: Supplementary file 1 [file Table_1.doc]

**Supplementary Table S1. PPI scores are shown for the interaction networks among the human proteins, including tPA, LRP1, PDGFR-ɑ, TIMP-1, TIMP-2, MMP-1, MMP-2, MMP-3, and MMP-**9.

| **Node1** | **Node2** | **Score** |
| --- | --- | --- |
| LRP1 | PLAT | 0.978 |
| PDGFRA | PLAT | 0.406 |
| PLG | PLAT | 0.948 |
| SERPINE1 | PLAT | 0.999 |
| TIMP-1 | PLAT | 0.537 |
| TIMP-2 | PLAT | 0.48 |
| MMP-1 | PLAT | 0.487 |
| MMP-14 | PLAT | 0.415 |
| MMP-2 | PLAT | 0.569 |
| MMP-3 | PLAT | 0.509 |
| MMP-9 | PLAT | 0.837 |
