# Supplementary material for: Beneficial Role of Rosuvastatin in Blood–Brain Barrier Damage Following Experimental Ischemic Stroke
Source: Front Pharmacol. 2018 Aug 21;9:926. doi: 10.3389/fphar.2018.00926 (PMC6110873; doi:10.3389/fphar.2018.00926)
Supplement: Supplementary file 2 [file Table_2.doc]

**Supplementary Table S2. The molecular docking of Rosu to PDGFR-α, LRP-1 and LDLr.** The affinity is ΔG. The inhibition constant for a drug against a target protein is K*i.*

| **Target** | **PDB (No.)** | **Rosuvastatin** | | |
| --- | --- | --- | --- | --- |
|  |  | **ΔG** | | **K*i* (nmol/l)** |
| PDGFR-α | 5K5X | -7.9 | 1758 | |
| LRP-1 | 2KNY | -6.7 | 13156 | |
| LDLr | 4NE9 | -8.2 | 1063 | |
