# Supplementary figures and images for: Beneficial Role of Rosuvastatin in Blood–Brain Barrier Damage Following Experimental Ischemic Stroke
Source: Front Pharmacol. 2018 Aug 21;9:926. doi: 10.3389/fphar.2018.00926 (PMC6110873; doi:10.3389/fphar.2018.00926)

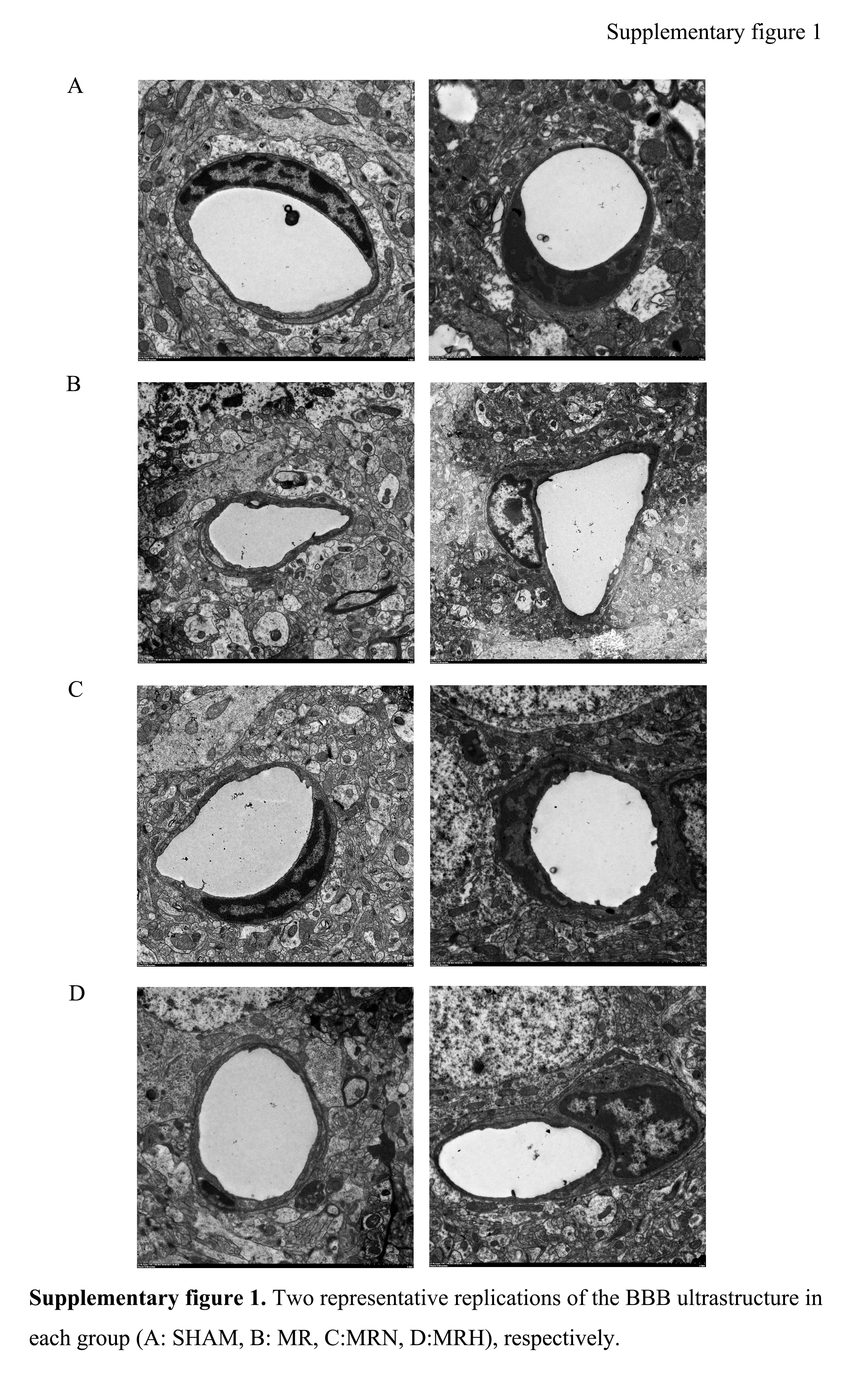

Supplement: Supplementary file 3 [file Image_1.JPEG]
